# Effect of chicory seed extract on glucose tolerance test (GTT) and metabolic profile in early and late stage diabetic rats

**DOI:** 10.1186/2008-2231-20-56

**Published:** 2012-10-15

**Authors:** Abdolreza Ghamarian, Mohammad Abdollahi, Xiaogang Su, Azita Amiri, Ali Ahadi, Azin Nowrouzi

**Affiliations:** 1 Department of Biochemistry, School of Medicine, Tehran University of Medical Sciences, Tehran, Iran; 2 Faculty of Pharmacy, and Pharmaceutical Sciences Research Center, Tehran University of Medical Sciences, Tehran, Iran; 3 University of Alabama School of Nursing, University of Alabama at Birmingham, Birmingham, AL, USA; 4 Department of Physiology, School of Medicine, Tehran University of Medical Sciences, Tehran, Iran; 5 Department of Clinical Laboratory Sciences, School of Allied Medical Sciences, Tehran University of Medical Sciences, Tehran, Iran

**Keywords:** Glucose tolerance test, Streptozotocin, Niacinamide, Chicory, Hyperglycemia

## Abstract

**Background and purpose of the study:**

The goal was to evaluate and compare the effects of aqueous extract of the seeds of chicory, *Cichorium intybus* L., on glucose tolerance test (GTT) and blood biochemical indices of experimentally-induced hyperglycemic rats.

**Methods:**

Late stage and early stage of Type 2 diabetes mellitus (T2DM) were induced in rats by streptozotocin (STZ) and a combination of STZ and niacinamide (NIA/STZ), respectively. Within each group, one subgroup received daily i. p. injections of chicory extract (125 mg/kg body weight, for 28 days). Body weight and fasting blood sugar (FBS) were measured weekly. Blood was analyzed for glycosylated hemoglobin (HbA1c) and sera for alanine aminotransferase (ALT), aspartate aminotransferase (AST), nitric oxide (NO), triacylglycerol (TG), total cholesterol (TC), total protein, and insulin on days 10 and 28 after treatment. Intraperitoneal glucose tolerance test (IPGTT) along with insulin determination was performed on a different set of rats in which the chicory-treated groups received the extract for 10 days.

**Results:**

During 4 weeks of treatment, chicory prevented body-weight loss and decreased FBS. ALT activities and levels of TG, TC and HbA1c decreased, and concentration of NO increased in the chicory treated groups (p < 0.05). Unlike late-stage diabetes, fasting serum insulin concentrations were higher and GTT pattern approximated to normal in chicory-treated early-stage diabetic rats.

**Conclusions:**

Chicory appeared to have short-term (about 2 hours, as far as GTT is concerned) and long-term (28 days, in this study) effects on diabetes. Chicory may be useful as a natural dietary supplement for slowing down the pace of diabetes progress, and delaying the development of its complications.

## Introduction

The number of individuals with Type 2 diabetes mellitus (T2DM) is increasing with a rate of three new cases every ten seconds (International Diabetes Federation's 5th edition of the Diabetes Atlas, 2011); and it is being diagnosed at younger age. Multiple risk factors behind the disease include chronic stress and depression [1,2], environmental pollutants and poisons [3], obesity and over-nutrition, and sedentary life style [4]; and the early onset may be exacerbated by Barker’s “Fetal origins of adult disease” hypothesis which calls to attention the impact on the offspring of the presence and severity of the risk factors in the pregnant mother [5].

Despite a multiplicity of drugs to affect proper blood glucose control [6], there is a tendency among the patients to seek additional remedy in traditional antidiabetic plant extracts. Medicinal plants appear to benefit diabetes in many ways [7,8]. *Cichorium intybus* L., commonly known as Chicory or Kasni (among Iranian folk), has been used in traditional medicine to treat a variety of diseases including high blood sugar [9-11]. Whole plant ethanolic extract was reported to decrease serum glucose in rats by reducing the activity of hepatic glucose 6-phosphatase [12]. Chicoric acid from chicory and basil leaves was found to enhance insulin release by pancreatic β cells and glucose uptake by muscle cells in culture [13]. Chicory has ameliorating effects against various liver toxicities [14-16] and oxidative stress [17]. Ethanolic seed extract rich in caffeoylquinic acid has recently been reported to improve glycemia in rats [18]; and a mixture of the aqueous extracts of six medicinal plants including chicory combined with stirred yoghurt filtrate was shown to protect against alloxan-induced oxidative stress and diabetes in rats [19]. However, chicory’s effects on early and late stage of diabetes have not been addressed and compared before.

Previously, we reported the effects of drinking intact chicory seed concoction on two diabetic patients. A 30% reduction in blood sugar (408 mg/dl to 286mg/dl) within one hour, and quick improvement in the state of fatigue and lethargy were observed in the patient who was on regular insulin (20 units Regular, 28 units NPH; daily) and metformin (two, daily), while it took 3 hours for the other patient who was not on drug or insulin therapy to experience the same percent decrease in blood sugar [20].

The present study was designed to investigate in more detail the differences between early and late stages of diabetes with regard to chicory’s effects on blood sugar and some other blood parameters that deviate in diabetes.

## Material and methods

### Materials

Chicory seeds were purchased from Bazar Bozorg Popular Market, Tehran, Iran and authenticated by Dr. Amin, a botanist of the Faculty of Pharmacy; Tehran University of Medical Sciences, with a voucher number PMP-710. STZ and NIA were purchased from Sigma (USA) and citrate buffer was obtained from Fluka (USA).

### Crude extract preparation

Chicory seeds were cleaned and powdered using an electric mill. Every 200 g was soaked in 1L of distilled water and refluxed for 20 minutes in a boiling water bath to make a 20% solution. The solution was allowed to cool at room temperature before being vacuum filtered through Watman No 1 filter paper. The filtrate was lyophilized at the Department of Physiology, School of Medicine, Tehran University of Medical Sciences, without further processing and stored at −20°C; every 100 g of powdered seed yielded about 8.2 g of lyophilized substance. The crude extract was not sterilized.

### Animals

All experiments were performed in the animal lab of the Faculty of Pharmacy with nine-week-old male Wistar-Albino rats weighing 200–250 g and with normal FBS levels around 86 ± 8 mg/dl. The animals were maintained in a temperature room (22 ± 2°C), with 12 h light/dark cycle and fed with standard rat chow and water *ad libitum*. Following several days of acclimatization, rats were randomly assigned to control and experimental groups. All ethical guidelines for use of laboratory animals were followed carefully.

The study involved three groups of rats (n = 28); one group of non-diabetic rats (Normal), and two groups of rats with diabetes; i.e., late-stage diabetic (STZ-diabetic rats), and early stage diabetic (NIA/STZ-diabetic rats). The study was ethically approved by TUMS (Tehran University of Medical Sciences) Review Board.

### Experimental diabetes induction

The two models of experimental diabetes were developed in rats after overnight fasting (8 h) as follows.

Late stage (advanced) diabetes was induced by a single dose of STZ (65mg/kg body weight) [21]; this group was called the “STZ” group. Early stage diabetes, which we called “NIA/STZ” group, was developed using a combination of STZ and niacinamide (NIA) according to modified Masiello method, where, an injection of STZ (65mg/kg body weight) was followed 15 minutes later, by an injection of niacinamide (NIA) (200mg/kg body weight) [22-24] in one step only. Both STZ and NIA were freshly prepared in 20 mM cold sodium citrate buffer solution (pH 4.5) and injected, intraperitoneally, with insulin needles.

Four days after injection, signs of severe and mild diabetes were detected, including bad smelling polyuria, polydipsia, aggressiveness, and restlessness. Diabetes induction was further confirmed by FBS measurement (Table 1). After confirmation of diabetes, each group was divided into, control and test, subgroups (n = 14) as shown in Table 1.

**Table 1:** A summary of the method: group divisions of rats, initial conditions and injections

**Group name**	**Initial**	**Initial**	**FBS (mg/dl)**	**IP Chicory Injection**
**(Sample size)**^**a**^	**Non-fasting BS (mg/dl)**	**Weight (g)**	**4 days after diabetes induction**	**(28 days)**
Control (10)	110.64 ± 5.62	200.36 ± 17.86	86.5 ± 7.17	-
Chicory-control (10)	104.14 ± 10.99	219.28 ± 15.42	87.64 ± 9.41	+
NIA/STZ (9)	111.35 ± 7.56	228.00 ± 13.94	137.78 ± 15.62	-
Chicory-NIA/STZ (10)	111.28 ± 6.82	231.71 ± 14.87	137.00 ± 29.39	+
STZ (7)	107.93 ± 11.29	213.71 ± 21.24	308.50 ± 117.7	-
Chicory-STZ (10)	110.92 ± 2.59	230.28 ± 16.24	330.14 ± 109.54	+

BS, blood sugar; FBS, fasting blood sugar; STZ, Streptozotocin, 65 mg/kg body weight; NIA, Niacinamide, 200 mg/kg body weight,15 min later; IP, intraperitoneal;. ^a^ refers to the number of animals on day 28 after treatment.

### Chicory treatment

Rats that were assigned to chicory-treated subgroups received daily i. p. injections of chicory (125 mg/kg body weight dissolved in 20-units volume of insulin syringe of normal saline) according to Pushparaj et al. [12] even though they had used whole plant ethanolic extract. The treatment started on the fifth day after induction of diabetes and lasted for 28 days. Table 1 provides a summary of group and subgroup divisions, initial weights, initial non-fasting blood sugar, and FBS after diabetes induction.

The dosage mentioned above was chosen also after a pilot experiment with limited numbers of STZ-treated rats (2 rats in two groups). Two doses of 40 and 100 mg/kg body weight were used. During 90 minute follow up of blood sugar in normally fed rats at a randomly chosen time of day (afternoon), the higher dose caused greater reduction of blood sugar. Blood sugar, (measured as explained below) decreased from 536 mg/dl down to 399 mg/dl, and from 510 mg/dl to 409 mg/dl with 100 and 40 mg/kg body weight of chicory extract, respectively. Normal saline and the previously measured quantity of the extract powder were mixed in Eppendorf tubes by a vortex mixer and injected immediately, to reduce the possibility of bacterial contamination. Despite many repeated i.p. injections, side effects, such as abdominal adhesions, were not observed in the treated rats.

### Weekly measurements

FBS was determined in a drop of blood from the tail using a glucometer (ACCU-CHECK, Roche, Germany) for four successive weeks. Likewise, body weights were recorded weekly, using a Triple Beam Balance, for later analysis and in order to adjust the weights of injected chicory extract powder according to the changing body weights.

### Blood sampling and analysis

Blood collection (fasting) was conducted twice: Once, 10 days after treatment with chicory, when only 4 members of each group were sacrificed; and a second time at the end of study time (28 days after the start of treatment with chicory extract) when the remaining 10 rats in each group were sacrificed. Rats were anesthetized using pentobarbital (40 mg/kg body weight dissolved in 50 ml normal saline; i. p. injection) and blood (3–4 ml) was taken from the heart. A sample (1ml) of the blood from each animal was collected in EDTA containing tubes (1.5 mg/ml) to be used for HbA1c measurement. The remaining blood samples were used to prepare sera that were stored at −80°C until used.

Fasting serum enzyme activities, ALT and AST, TG, TC and total protein concentrations in blood samples from days 10 and 28 were measured in Shariati Hospital Pathology Lab by their respective kits (from Human diagnostics, Germany) and HbA1c% was also determined by kit (RANDOX, UK), using an automatic analyzer (Hitachi 902, Japan-Germany). Insulin was determined by Rat Insulin ELISA Kit according to the manufacturer’s instructions (Mercodia, Sweden). Biochemical assay results have been presented only for the final day of study (day 28) due to similarity of the results (Table 2).

**Table 2:** Summary statistics of blood variables values after 28 days of i.p. chicory extract injection

**Measured parameter**	**Control**	**Chicory-control**	**NIA/STZ**	**Chicory-NIA/STZ**	**STZ**	**Chicory-STZ**
Weight	227.3 ± 15.2	247.6 ± 28.8	218.1 ± 21.2	242.6 ± 14.1	186.8 ± 13.7	229.9 ± 25.7
*AST	119.1 ± 20.1	128.6 ± 20.1	124.6 ± 28.6	126.0 ± 47.6	151.7 ± 64.9	103.7 ± 20.4
*ALT^a†,b†,c‡,f†^	43.7 ± 9.9	47.4 ± 11.9	46.2 ± 7.5	40.6 ± 9.0	87.8 ± 43.0	51.2 ± 15.7
TG^a‡,b†^	78.5 ± 31.7	83.3 ± 27.1	111.2 ± 38.1	82.8 ± 44.7	121.6 ± 26.9	97.3 ± 33.5
TC^a†,b†d#^	40.6 ± 9.9	43.0 ± 6.9	48.3 ± 7.7	51.3 ± 11.0	56.2 ± 9.6	49.6 ± 7.9
Total protein	6.10 ± 0.5	5.9 ± 0.2	6.0 ± 0.2	6.4 ± 0.6	5.8 ± 0.5	5.7 ± 0.5
HbA1c^aŦ,bŦ, d†,e†,fŦ^	3.9 ± 0.2	3.9 ± 0.2	4.0 ± 0.4	4.0 ± 0.1	8.0 ± 2.3	6.5 ± 1.9
NO^a‡, b#,c‡^	37.0 ± 4.6	37.0 ± 5.0	34.46.0	36.8 ± 4.0	31.2 ± 5.1	38.3 ± 6.4
FBS	77.7 ± 6.6	77.1 ± 3.0	159.2 ± 7.6	110.4 ± 8.5	416.5 ± 33.0	361 ± 86.1
Insulin	2.5 ± 0.4	2.5 ± 0.8	0.08 ± 0.3	0.8 ± 0.3	NA	NA

Weight (gram), AST and ALT, specific activities of aspartate amino transferase and alanine amino transferase, (Units per grams protein); TG, triacylglycerol (mg/dl); TC, total cholesterol (mg/dl); Total protein (g/dl); HbA1c, glycosylated hemoglobin (%); and NO, nitric oxide (μmol/dl), FBS, fasting blood sugar (mg/dl); Insulin (μg/l). Data are presented as mean ± SD. ^NA^ Insulin concentrations were considered undetectable, as the values were negative −0.76 ± 0.3 and −0.66 ± 0.4 for CS and TCS, respectively. i.p, intraperitoneal;* AST and ALT were adjusted by a division of Total protein values. ^a^control vs. STZ; ^b^chicory-control vs. STZ; ^c^STZ vs. chicory-STZ; ^d^control vs. chicory-STZ; ^e^chicory-control vs. chicory-STZ, ^f^STZ vs. NIA/STZ; ^†^p < 0.01; ^‡^p < 0.05; ^Ŧ^p < 0.001; ^#^p ≤ 0.05.

### Nitric oxide determination

NO was measured by modified Griess reaction [25]. Briefly, serially diluted standards or deproteinated serum samples, 2% sulfanilamide in 1M HCl, 0.1% *N*-(1-Naphthyl)ethylenediamine in ddH_2_O, and then vanadium chloride solution (80 mg dissolved in 10 ml of 1M HCl) were mixed (50 μl of each) in the mentioned order in a 96-well plate. After 45 min of incubation at 37°C, optical density was determined at 450 nm by a microplate reader (2020 Anthos, Austria). NO concentrations in the samples were determined using a standard calibration curve. Deproteination of serum samples was accomplished by 3000 MWCO VIVASPIN filters from Sartorius (Germany).

### Intraperitoneal glucose tolerance test (IPGTT)

Another set of rats (5 groups also named control, STZ, NIA/STZ, chicory-STZ, and chicory-NIA/STZ, consisting of 6 rats each and prepared as explained in section of experimental diabetes above) was subjected to IPGTT. A chicory-control group was not included in this section of the study due to insufficient Rat Insulin Elisa Kit. Briefly, after an overnight fast, FBS was measured in six normal rats (control). The rats were anaesthetized; glucose (2 g/body weight) together with lyophilized extract of chicory (125 mg/body weight) were dissolved in 30-units volume of normal saline and injected intraperitoneally. Blood glucose was determined every 30 minutes in a drop of blood from the tail; at the same time, whole blood (1ml, every 30 min) was taken from the heart and sera were prepared for insulin measurement. The same procedure was conducted on two other groups of rats, STZ and NIA/STZ, immediately after being made diabetic. Because blood analysis on day 10 showed similar trend in the changes of blood parameters as day 28, rats in groups chicory-STZ and chicory-NIA/STZ were treated with chicory extract for 10 days prior to IPGTT.

### Statistical analysis

The collected data essentially involved a 6 × 6 repeated measures with treatment groups as between-subjects factors and days of study as within-subjects factors. Data for weight and FBS were summarized with mean ± SD. Linear mixed effects models (SAS PROC MIXED) were used as the primary tool for assessment followed by a post-hoc least significance difference (LSD) for pair wise comparisons. Minor imputation was done for missing values. We assumed the unstructured covariance matrix to carry out the analysis. Akaike's Information Criterion (AIC) and Schwarz' Bayesian Criterion (SBC) were used to determine the underlying covariance structure. According to AIC, the autoregressive (AR1) of order 1 was used to model the covariance structure of longitudinal measures on each rat in the linear mixed effect model. Data of the blood variables on days 28 were presented as mean ± SD.

For the analysis of the 120 minutes follow-up data on blood glucose and insulin, truncated polynomial spline basis terms were introduced to model the nonlinearity and change points seen in the longitudinal progression for these measures. The area under curve (AUC) was determined by Image J Software (NIH) and compared using Kruskal-Wallis Test (nonparametric ANOVA). A p-value lower than 0.05 was considered statistically significant.

## Results

### Blood sugar after diabetes induction

Although non-fasting blood sugar was measured at time zero, it can be inferred from following the FBS of non-diabetic groups (control and chicory-control) that normal FBS is around 86.5 ± 7.17 to 87.64 ± 9.41 (Table 1). In late-stage diabetic rat groups (STZ and chicory-STZ), a single STZ injection increased FBS about 3.5-fold. When one STZ injection was followed by a single injection of niacinamide (NIA), rats developed a form of early stage diabetes mellitus and FBS increased nearly 1.8-fold in NIA/STZ and chicory-NIA/STZ groups.

### Weekly measurement of body weight

**Figure 1:**
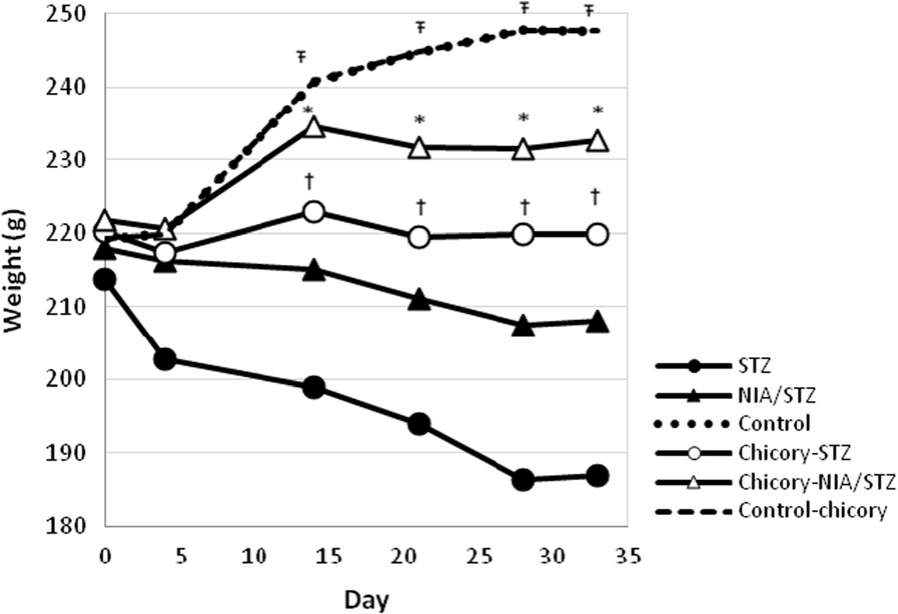
Mean weight by day for each group.

During the 4-wk treatment with chicory, the weight in control and chicory-control groups increased significantly (about 13%) as a result of ordinary growing up. As shown in Figure 1, the weight loss was significant (12.5%) in STZ group and negligible in NIA/STZ group (4.5%). The mean weight remained nearly constant during the study time in chicory-STZ group (1.6% weight losses only). An overall comparison of the groups across all time points suggested that chicory helped prevent excessive weight loss in both early- and late-stage diabetes mellitus, yet it did not lead to a weight gain either since chicory extract administration to normal healthy rats did not encourage any increase in weight (chicory-control compared with control).

Chicory treatment prevented excessive weight loss in both late and early stage of diabetes according to an overall comparison STZ with chicory-STZ (one sided p < 0.05) and NIA/STZ with chicory-NIA/STZ (one sided p < 0.01) across all time points. Ŧ show statistically significant differences between both control and chicory-control with all other groups; *show significant difference between chicory-STZ and STZ-diabetic groups; †show significant differences between chicory-NIA/STZ and NIA/STZ. Treatment with chicory started on day 5 after STZ and STZ + NIA injections and lasted for 28 days, thus a total of 33 days of study.

### Weekly measurements of FBS

**Figure 2:**
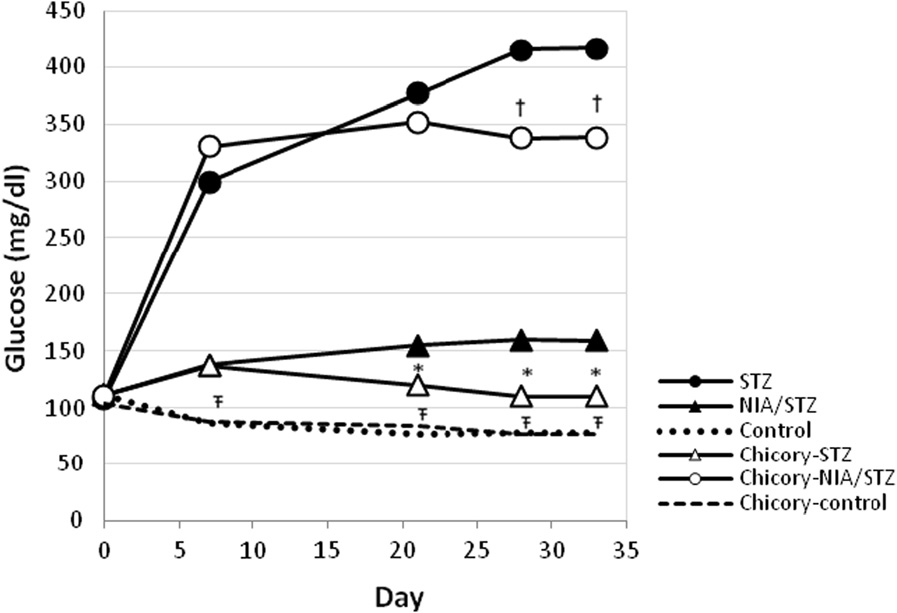
Mean FBS by day for each group.

FBS levels did not change in control and chicory-control groups during 4-wk study time. FBS increased drastically in STZ and moderately, but significantly, in NIA/STZ groups. Chicory treated groups, chicory-STZ and chicory-NIA/STZ, were able to resist excessive increases in FBS (Figure 2). An overall comparison of the groups across all time points indicated chicory’s ability to lower blood sugar in both severe and mild diabetic rats. Nonetheless, on the final day of study, FBS was still much higher in chicory-STZ (final day vs. initial day: p-value <0.0001).

The level of FBS remained mainly unchanged for control and chicory-control groups, increased considerably in STZ and moderately in NIA/STZ diabetic rats. Treatment with chicory extract lowered fasting blood sugar level in STZ (late stage) and NIA/STZ (early stage) diabetic rats (one-sided p < 0.05 for STZ vs. chicory-STZ and NIA/STZ vs. chicory-NIA/STZ groups). Body weight was included as a covariate to reduce variability. Ŧ show statistically significant differences between both control and chicory-control with all other groups; *show significant difference between chicory-STZ and STZ-diabetic groups; † show significant differences between chicory-NIA/STZ and NIA/STZ. Treatment with chicory started on day 5 after STZ and STZ + NIA injections and lasted for 28 days, thus a total of 33 days of study.

### Blood parameters at the end of 4-wk study time

The results are summarized in Table 2. In STZ-diabetic group, 4 wk after diabetes induction, significant increases in ALT, TG, TC, HbA1c, and FBS and a significant decrease in NO comprised the major complications. In chicory-STZ group, daily chicory administration for 28 days, led to improvement of conditions even though HbA1c and FBS were still higher than normal. In NIA/STZ, the main variations included slight increases in TG and TC. Over a 4 wk treatment with chicory extract, the value of TG decreased toward normal. Total cholesterol increased slightly further after treatment with chicory for 28 days despite a decrease on day 10 after treatment.

### IPGTT

According to Table 2, the effects of chicory on FBS and Insulin were most obvious in NIA/STZ-diabetic rats. In NIA/STZ, the remaining beta cells of the pancreas were still producing some insulin, which despite being significantly lower than normal (control vs. NIA/STZ, p < 0.001) was keeping blood sugar concentration at around 150 mg/dl. After treatment with chicory, insulin levels increased in chicory-NIA/STZ (chicory-NIA/STZ vs. NIA/STZ, p < 0.001 without and p < 0.05 with adjustment for weight).

**Figure 3:**
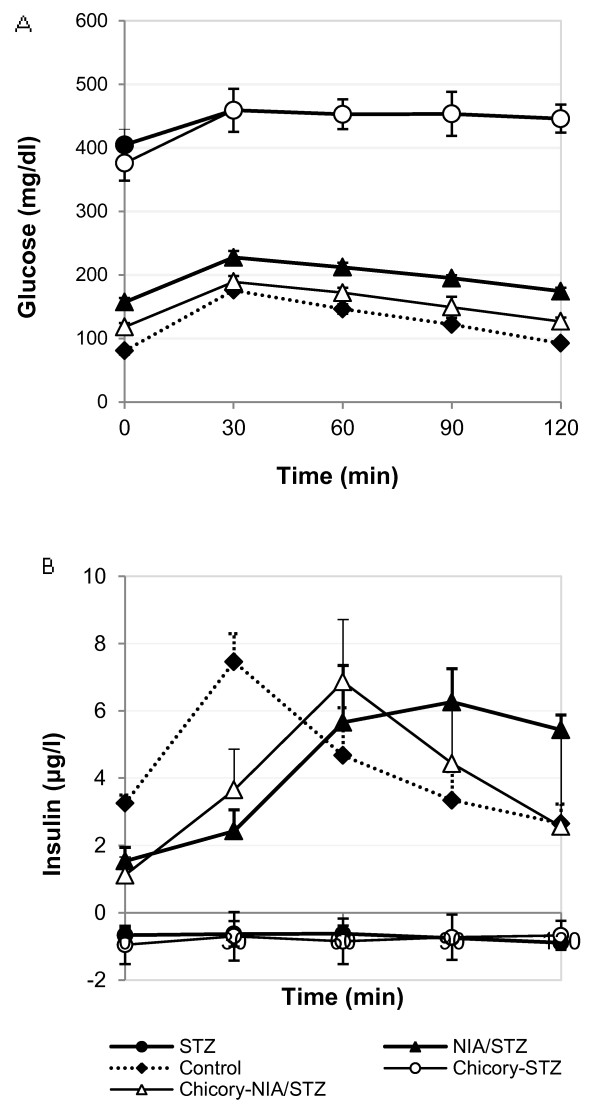
**IPGTT.** Mean plots for blood glucose (**A**) and insulin (**B**) by time. (**A**) Blood glucose follow-up for 120 minutes. Blood sugar levels in NIA/STZ were statistically and significantly higher than control at all points in time during GTT (p < 0.0001). Chicory lowered blood sugar values closer to control. (*Control vs. chicory-NIA/STZ*: initial, p <0.0001; 30 min, p > 0.05; 60 min, p = 0.01; 90 min, p = 0.05; 120 min, p < 0.001. *NIA/STZ vs. chicory-NIA/STZ*: initial, p < 0.001; 30 min, p < 0.01; 60 min, p < 0.001; 90 min, p < 0.01; 120 min, p < 0.0001). (**B**) Blood insulin follow-up for 120 min. The timing of maximum insulin was 30 min, 60 min and 90 min for control, chicory-NIA/STZ, and NIA/STZ, respectively.

The mean plots for blood glucose and insulin demonstrate the progression patterns across different groups (Figure 3A and B). Blood glucose levels stayed high and insulin was undetectable in STZ and chicory-STZ groups during the 120-minute follow up. In chicory-NIA/STZ group, blood sugar values were lower and so close to control group that occasionally, at 30 and 90 min, the difference between control and chicory-NIA/STZ became insignificant statistically. However, according to AUC, total glucose showed a significant difference between control and NIA/STZ groups (P<0.001); but the AUCs for NIA/STZ and chicory-NIA/STZ groups were not significantly different (p>0.05).

According to Figure 3B, there was a significant difference regarding the progression nonlinear patterns among Control, NIA/STZ, and chicory-NIA/STZ groups (p < 0.001). In terms of the change point analysis, it could be confirmed that Control had a peak or change point at min 30 (p < 0.0001); chicory-NIA/STZ had a change point at min 60 (p < 0.0001); and NIA/STZ had a peak or change point at min 90 (p < 0.001). AUC calculation for insulin showed that total insulin levels were not significantly different in three groups (Table 3).

**Table 3:** **Area under curve (AUC) for glucose and insulin in Figure**3

**Area Under Curve (AUC)**	**Control**	**NIA/STZ**	**Chicory-NIA/STZ**	**STZ**	**Chicory-STZ**
Glucose	156524 ± 4099	178229 ± 6595	172765 ± 3354	195472 ± 2408	190161 ± 3828
Insulin	128189 ±10450	143380 ± 7985	123864 ± 7024	NA	NA

AUC for glucose in control was significantly different than AUC for NIA/STZ (P<0.001). AUCs for insulin were not significantly different among the three groups, p > 0.05). NA, non applicable.

## Discussion

Early stage T2DM transformation to advance diabetes occurs over time and is due to gradual destruction of pancreatic β cells. As prevention is meant to inhibit an abnormal condition from getting worse or to delay the side effects of a disease, it seemed important to us to investigate the effects of chicory seed extract on early diabetes.

### IPGTT

The effect of chicory extract on GTT was the main observation of this study, which is summarized in Figures 3A and B. The comparison of GTT plots for Control, NIA/STZ, and chicory-NIA/STZ groups may suggest a relative improvement of insulin sensitivity and a reduction of insulin resistance in the chicory-treated group (chicory-NIA/STZ) (Figure 3B). Hyperinsulinemia in NIA/STZ implies that peripheral tissue insulin resistance occurred after partial beta cell destruction, while the alleviation of hyperinsulinemia together with the alleviation of ALT activity in the chicory-treated group suggests an amelioration of insulin resistance [26]. The change in the peak insulin time point in chicory-NIA/STZ compared to NIA/STZ (60 min vs. 90 min) is an indication of a quicker response of the pancreatic beta cells to postprandial hyperglycemia and further supports chicory’s ability to stimulate insulin secretion form pancreatic beta cells. The same effects are not observed in chicory-STZ group presumably because of lack of insulin in advanced diabetes. It can be argued that chicory plays the role of an insulin-sensitizer and should also be able to act in late stage diabetic patients who receive exogenous insulin injections.

### Blood parameters

Lack of insulin in chicory-STZ, however, did not stop chicory’s long-term positive effects on blood parameters. Deaths were prevented in chicory treated severe diabetes. The decrease in TG and TC in the blood of chicory-STZ rats may be secondary to improved lipid metabolism [27] and inhibition of adipogenesis [10]. In chicory-NIA/STZ rats, the increase in TC over 28 days of exposure to chicory extract may be due to an increase in HDL-cholesterol according to Kim and Shin [27].

In our study, NO levels were found to be low in the blood of diabetic rats. Several studies have shown that patients with Type 2 diabetes have decreased endothelial nitric oxide (eNO) production, and enhanced plasma concentrations of asymmetrical dimethylarginine (ADMA), an inhibitor that suppresses eNO synthesis and produces vascular dysfunction and neurological disorders [28]. As NO is also an important player in the nervous system, there may be a beneficial role of chicory in the prevention of both cardiovascular and neurological implications of diabetes, as it has been described for the aging myocardium of albino rats and oxidative defense system in the brain of diabetic rats [29,30].

The rise in the activity of liver enzymes ALT and AST in blood is common in liver diseases; but several recent studies have suggested the circulating ALT levels at the upper range of normal limits and high ALT/AST ratios to be associated with high prevalence of metabolic syndrome, insulin resistance (IR) and obesity [26,31-33]. In this study, severe diabetes was accompanied by statistically significant increase in ALT specific activities that was lowered by chicory treatment, a confirmation of the hepatoprotective properties of chicory and improvement of metabolic syndrome and insulin resistance. AST specific activity and Total protein levels were sustained according to Table 2, the former implying higher ALT/AST ratios.

Glycosylated hemoglobin (HbA1c) that had almost doubled in STZ rats indicated some reduction in the chicory-treated late stage diabetes (chicory-STZ) meaning that blood sugar levels were kept at relatively lower levels under the influence of chicory extract. The observations related to blood parameters in severe diabetes suggest that chicory may be also capable to act through insulin-independent mechanisms.

Obesity is a risk factor for developing diabetes mellitus; but diabetes itself is accompanied by loss of weight due to muscle wasting. Diabetic patients suffer from negative nitrogen balance [34]. Chicory extract prevented weight loss in both severe and mild diabetes. Weight loss inhibition could be due to an easing of glucose transportation into the peripheral tissue as a result of possible correction of insulin resistance (in early diabetes) and improved lipid metabolism (in late stage diabetes); it cannot be ruled out, however, that chicory extract components may also have further effects directly on protein metabolism [35].

Unlike the bitter taste of chicory leaves and roots, chicory seed decoction is palatable. Although many studies have used alcoholic extract of chicory, homemade preparations are usually made in water, as alcohol is both toxic to the liver and prohibited in many cultures. The glucose lowering capacity of chicory has been attributed to its chemical composition including antioxidant compounds [17], anthocyanins [36], tannins [10], coumarins, chicoric acid, chlorogenic acid, and caffeic acid [37]. The role of antioxidants in the management of diabetes and its complications has been reviewed [38]. Chicory possesses a strong antioxidant capacity; for the aqueous seed extract used in this study, 1, 1-diphenyl-2-picryl hydrazyl radical (DPPH)-50% inhibition (IC50) was calculated to be 180.5 μg/ml by DPPH radical-scavenging assay [39]. Chicory contains vitamins and many of the essential amino acids, and carbohydrates [9]. The effects of chicory seed extract observed in this study are despite the presence of sugar in the extract (total of approximately 183.7 mg soluble sugar per g dry weight of stock powder, measured by Kochert method [40]). The sugar in the seed extract includes sucrose, and an oligosaccharide which was shown by chromatographic analysis to consist of glucose and fructose (data not shown). The quantity of glucose, fructose and sucrose has been measured by Jurgonskiet al [41]; they also examined water-soluble and lipid-soluble fractions of chicory peel and seed extract for antioxidant capacity and their effects on the activities of superoxide dismutase and glutathione peroxidase enzymes [18,41].

## Conclusions

The results of the present study suggest that chicory may exert both short- and long-term effects on diabetes, the former being under the influence of insulin and through its insulin-sensitizing action. The long-term beneficial effects of chicory may arise without the assistance of insulin.

## Competing interests

There are no conflicts of interests related to this publication.

## Authors' contributions

GA carried out the practical work; AA was a graduate committee member and research advisor; SX performed the statistical analysis; AA helped in manuscript preparation. AA assisted with plant extract preparation; NA designed, supervised/reviewed the entire study and wrote the manuscript. All authors read and approved the final manuscript.
